# A dynamical study of pulse-coupled oscillators in the brain

**DOI:** 10.1186/1471-2202-13-S1-O12

**Published:** 2012-07-16

**Authors:** Tanushree Luke, Ernest Barreto, Paul So

**Affiliations:** 1 School of Physics, Astronomy,& Computational Sciences, George Mason University, Fairfax, VA 22030, USA; 2 The Krasnow Institute for Advanced Study, George Mason University, Fairfax, VA 22030, USA

## 

In 1967, Winfree [1] proposed a novel mathematical approach to describe phenomena of collective synchrony in nature (i.e. flashing of fireflies, clapping in a theatre, alpha rhythms, etc.) using a large coupled network of phase oscillators with a diversity of natural frequencies. By analyzing this large heterogeneous network from a “mean field” approach, the spontaneous synchrony can be understood as a critical phase transition similar to most statistical mechanical systems.

In this work, we employ this approach to model the phase transitions and bifurcation structures of a large network of pulse-coupled theta neurons [2] by appropriate choice of Winfree's "response" and "influence" functions, the latter of which is parameterized by a "sharpness" parameter *n *[1]. As this parameter increases, the influence function approximates the behavior of a pulse-coupled synapse. Assuming a Lorentzian distribution of natural frequencies of width *Δ* and mean value *ω_0_*, taking the thermodynamic limit, and employing the Ott-Antonsen reduction method [3], the collective dynamics of the pulse-coupled network can be analytically reduced to a single low-dimensional dynamical equation for the mean field parameter *z*(*t*).

We analyze the bifurcation diagrams for different values of the sharpness parameter *n*. We find that more complex behavior is apparent with increasing sharpness of the influence function, and that equilibria outside the physically relevant region (within the unit circle) affect the transient dynamics of *z*(*t*) inside the circle. Further, we find that some level of coherence always exists in the network for non-zero coupling, in contrast to other mean field coupled phase oscillator networks [1,4]. Most interestingly, heterogeneity is observed to suppress complexity in the collective/macroscopic behavior. As the network becomes more homogeneous (∆→0), more complex dynamic including aperiodic and multistability emerge from the macroscopic mean field.

**Figure 1:**
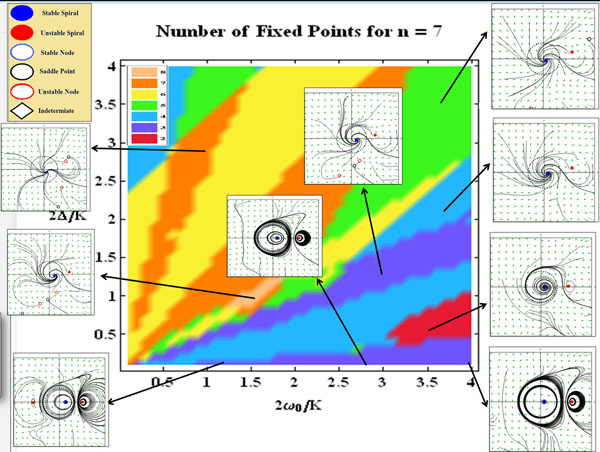
A sample bifurcation diagram showing the complex structure of fixed points at various locations in parameter space, for a sharpness parameter of (n = 7). Several representative phase portraits from several distinct region of parameter space are included.
